# Ternary structure of *Plasmodium vivaxN*-myristoyltransferase with myristoyl-CoA and inhibitor IMP-0001173

**DOI:** 10.1107/S2053230X24008604

**Published:** 2024-09-18

**Authors:** Cydni Bolling, Alex Mendez, Shane Taylor, Stanley Makumire, Alexandra Reers, Rachael Zigweid, Sandhya Subramanian, David M. Dranow, Bart Staker, Thomas E. Edwards, Edward W. Tate, Andrew S. Bell, Peter J. Myler, Oluwatoyin A. Asojo, Graham Chakafana

**Affiliations:** a Chemistry and Biochemistry Department Hampton University 200 William R. Harvey Way Hampton VA23668 USA; b Structural Biology Research Unit, Faculty of Biochemistry and Molecular Medicine University of Oulu Aapistie 7C 90220Oulu Finland; c Seattle Structural Genomics Center for Infectious Diseases, Seattle, Washington, USA; d Center for Global Infectious Disease Research Seattle Children’s Research Institute 307 Westlake Avenue North Suite 500 Seattle WA98109 USA; e UCB BioSciences, Bainbridge Island, WA98110, USA; f Imperial College London South Kensington Campus LondonSW7 2AZ United Kingdom; g Myricx Pharma, 125 Wood Street, LondonEC2V 7AN, United Kingdom; h Dartmouth Cancer Center Dartmouth College One Medical Center Drive Lebanon NH03756 USA; University of York, United Kingdom

**Keywords:** *N*-myristoyltransferases, *Plasmodium vivax*, malaria, G6PD deficiency

## Abstract

*Plasmodium vivax* is a major cause of malaria, which poses an increased health burden on approximately one third of the world’s population due to climate change. Primaquine, the preferred treatment for *P. vivax* malaria, is contraindicated in individuals with glucose-6-phosphate dehydrogenase (G6PD) deficiency, a common genetic cause of hemolytic anemia, that affects ∼2.5% of the world’s population and ∼8% of the population in areas of the world where *P. vivax* malaria is endemic. The Seattle Structural Genomics Center for Infectious Disease (SSGCID) conducted a structure–function analysis of *P. vivaxN*-myristoyltransferase (*Pv*NMT) as part of efforts to develop alternative malaria drugs. *Pv*NMT catalyzes the attachment of myristate to the N-terminal glycine of many proteins, and this critical post-translational modification is required for the survival of *P. vivax*. The first step is the formation of a *Pv*NMT–myristoyl–CoA binary complex that can bind to peptides. Understanding how inhibitors prevent protein binding will facilitate the development of *Pv*NMT as a viable drug target. NMTs are secreted in all life stages of malarial parasites, making them attractive targets, unlike current antimalarials that are only effective during the plasmodial erythrocytic stages. The 2.3 Å resolution crystal structure of the ternary complex of *Pv*NMT with myristoyl-CoA and a novel inhibitor is reported. One asymmetric unit contains two monomers. The structure reveals notable differences between the *Pv*NMT and human enzymes and similarities to other plasmodial NMTs that can be exploited to develop new antimalarials.

## Abstract 2

The 2.3 Å resolution ternary structure of the essential *P. vivaxN*-myristoyltransferase with myristoyl-CoA and a peptide-binding domain inhibitor is reported as part of ongoing efforts by the SSGCID for the rational design of new therapeutics for malaria.

## 1. Introduction

Malaria, a deadly disease caused by protozoan parasites from the *Plasmodium* genus, poses a significant global health challenge. *P. vivax* is responsible for the most widespread human malaria and is an obstacle to global malaria-elimination efforts, with nearly 2.5 billion people, or more than one-third of the world’s population, at risk of *P. vivax* infection (Battle *et al.*, 2019 ▸). The dormant liver phase of *P. vivax* enables its survival in colder climates and tropical, subtropical and temperate regions, giving a wider geographical range (Battle *et al.*, 2019 ▸). Primaquine is the most effective drug for *P. vivax* infection, but low adherence lowers its efficacy (Mehdipour *et al.*, 2023 ▸). Furthermore, primaquine is contraindicated among individuals with glucose-6-phosphate dehydrogenase (G6PD) deficiency, which causes hemolytic anemia and other complications (Kane, 2012 ▸; Yilma *et al.*, 2023 ▸). This is particularly concerning since approximately 400 million people worldwide have G6PD deficiency (Drysdale *et al.*, 2023 ▸). There is therefore a pressing need for safer treatment options for *P. vivax* malaria.

A promising strategy for the eradication of malaria is targeting proteins that regulate multiple stages of the life cycle of *Plasmodium* species. One such protein, *N*-myristoyltransferase (*Pv*NMT), is an essential enzyme that catalyzes a post-translational modification (myristoylation) through transfer of the lipid myristate from myristoyl coenzyme A (Myr-CoA) to the N-terminal glycine residues of target proteins (Selvakumar *et al.*, 2011 ▸; Udenwobele *et al.*, 2017 ▸; McIlhinney, 1989 ▸). NMT-mediated myristoylation is crucial for membrane association, protein–protein interactions, protein stability and turnover, and signal transduction (Selvakumar *et al.*, 2011 ▸). NMTs also help to regulate cellular processes and have emerged as potential therapeutic targets for parasitic diseases (Frearson *et al.*, 2010 ▸; Rodríguez-Hernández *et al.*, 2023 ▸; Harupa *et al.*, 2020 ▸).

NMTs have been explored as potential drug targets against *Plasmodium* parasites and are promising for the development of innovative therapeutic approaches to combat malaria (Rackham *et al.*, 2014 ▸; Nicolau *et al.*, 2023 ▸; Rodríguez-Hernández *et al.*, 2023 ▸). *Plasmodium* species possess a single NMT gene that is essential for survival, and previous studies showed reduced parasitemia after NMT inhibition (Pino *et al.*, 2012 ▸). An advantage of targeting NMT is that it is expressed throughout every stage of the life cycle of *Plasmodium* species, which allows the possibility of complete parasite elimination, unlike many licensed antimalarials, which only target the erythrocytic stage (Pino *et al.*, 2012 ▸). A recent study also demonstrated that *Pv*NMT inhibitors reduced parasite growth in the schizont and hypnozoite stages, during which several essential NMT substrates are expressed (Rodríguez-Hernández *et al.*, 2023 ▸). Our study presents the 2.3 Å resolution crystal structure of *Pv*NMT bound to Myr-CoA and a novel inhibitor, contributing to the quest for alternative drug treatments against malaria using *Pv*NMT as a target (Harupa *et al.*, 2020 ▸; Rodríguez-Hernández *et al.*, 2023 ▸).

## 2. Materials and methods

### 2.1. Macromolecule

The gene (*Pv*NMT; UniProt A5K1A2) encoding amino acids 1–410 was acquired from GenScript as a synthetic construct inserted into pET-11a, encoding a 3C protease-cleavable hexahistidine tag (MGSSHHHHHHSAALEVLFQGP-ORF; Table 1 ▸). *Pv*NMT was expressed and purified using established protocols (Stacy *et al.*, 2011 ▸; Serbzhinskiy *et al.*, 2015 ▸; Rodríguez-Hernández *et al.*, 2023 ▸). Plasmid DNA was transformed into chemically competent *Escherichia coli* BL21(DE3) Rosetta cells. The plasmid containing His-*Pv*NMT was tested for expression and 2 l of culture was grown using auto-induction medium (Studier, 2005 ▸) in a LEX Bio­reactor (Epiphyte Three) as described previously (Serbzhinskiy *et al.*, 2015 ▸). The expression clone can be requested online at https://www.ssgcid.org/available-materials/expression-clones/.

*Pv*NMT was purified in two steps: immobilized metal (Ni^2+^) affinity chromatography (IMAC) and size-exclusion chromatography (SEC) on an ÄKTApurifier 10 (GE Healthcare) using automated IMAC and SEC programs (Serbzhinskiy *et al.*, 2015 ▸). Briefly, thawed bacterial pellets (25 g) were lysed by sonication in 200 ml lysis buffer [25 m*M* HEPES pH 7.0, 500 m*M* NaCl, 5%(*v*/*v*) glycerol, 0.5%(*w*/*v*) CHAPS, 30 m*M* imidazole, 10 m*M* MgCl_2_, 1 m*M* TCEP and five tablets of protease-inhibitor cocktail (cOmplete Mini, EDTA-free Roche, Basel, Switzerland)]. After sonication, the crude lysate was clarified with 20 µl (25 units ml^−1^) of Benzonase by incubating and mixing at room temperature for 45 min. The lysate was clarified by centrifugation at 5000*g* for 1 h at 277 K using a refrigerated Sorvall centrifuge (Thermo Scientific). The clarified supernatant was then passed over a 5 ml Ni–NTA HisTrap FF column (GE Healthcare) which had been pre-equilibrated with loading buffer [25 m*M* HEPES pH 7.0, 500 m*M* NaCl, 5%(*v*/*v*) glycerol, 30 m*M* imidazole, 1 m*M* TCEP, 0.025%(*w*/*v*) sodium azide]. The column was washed with 20 column volumes (CV) of loading buffer and eluted with elution buffer [25 m*M* HEPES pH 7.0, 500 m*M* NaCl, 5%(*v*/*v*) glycerol, 30 m*M* imidazole, 1 m*M* TCEP, 0.025%(*w*/*v*) sodium azide, 250 m*M* imidazole] over a 7 CV linear gradient. Peak fractions were pooled, concentrated to 5 ml and loaded onto a Superdex 75 26/60 column (GE Biosciences) equilibrated with running buffer (20 m*M* HEPES pH 7.0, 300 m*M* NaCl, 5% glycerol, 1 m*M* TCEP). *Pv*NMT eluted from the SEC column as a single, monodisperse symmetrical peak that accounted for >90% of the protein product, with a molecular mass of ∼40 kDa, suggesting purification as a monomer (based on the theoretical molecular weight of 47.1 kDa). The pure peak fractions were pooled and concentrated to 13.5 mg ml^−1^ using an Amicon purification system (Millipore). The purified protein was stored in 100 µl aliquots at 193 K and can be requested online at https://www.ssgcid.org/available-materials/ssgcid-proteins/.

### 2.2. Crystallization

*Pv*NMT was crystallized at 290 K in sitting-drop vapor-diffusion format. Briefly, 13.5 mg ml^−1^ protein was incubated with final concentrations of 0.4 m*M* Myr-CoA and 0.4 m*M* IMP-0001173 at 4°C for 30 min and then mixed in a 1:1 ratio with precipitant solution as described in Table 2 ▸. Before data collection, the crystals were harvested and cryoprotected with 20%(*v*/*v*) ethylene glycol (Table 2 ▸).

### 2.3. Data collection and processing

Data were collected at 100 K on beamline 5.0.2 at the Advanced Light Source (ALS), Lawrence Berkeley National Laboratory (Table 3 ▸). Data were integrated with *XDS* and reduced with *XSCALE* (Kabsch, 2010 ▸). Raw X-ray diffraction images have been stored at the Integrated Resource for Reproducibility in Macromolecular Crystallography at https://www.proteindiffraction.org.

### 2.4. Structure solution and refinement

The structure of *Pv*NMT was determined by molecular replacement with *Phaser* (McCoy *et al.*, 2007 ▸) from the *CCP*4 suite of programs (Collaborative Computational Project, Number 4, 1994 ▸; Krissinel *et al.*, 2004 ▸; Winn *et al.*, 2011 ▸; Agirre *et al.*, 2023 ▸) using PDB entry 5v0x as the search model. The structure quality was checked using *MolProbity* (Williams *et al.*, 2018 ▸). Electron-density maps showing the ligand fit are shown in Supplementary Fig. S1. Data-reduction and refinement statistics are shown in Table 4 ▸. Coordinate and structure factors have been deposited with the Worldwide PDB (wwPDB) as entry 6b1l.

## 3. Results and discussion

**Figure 1:**
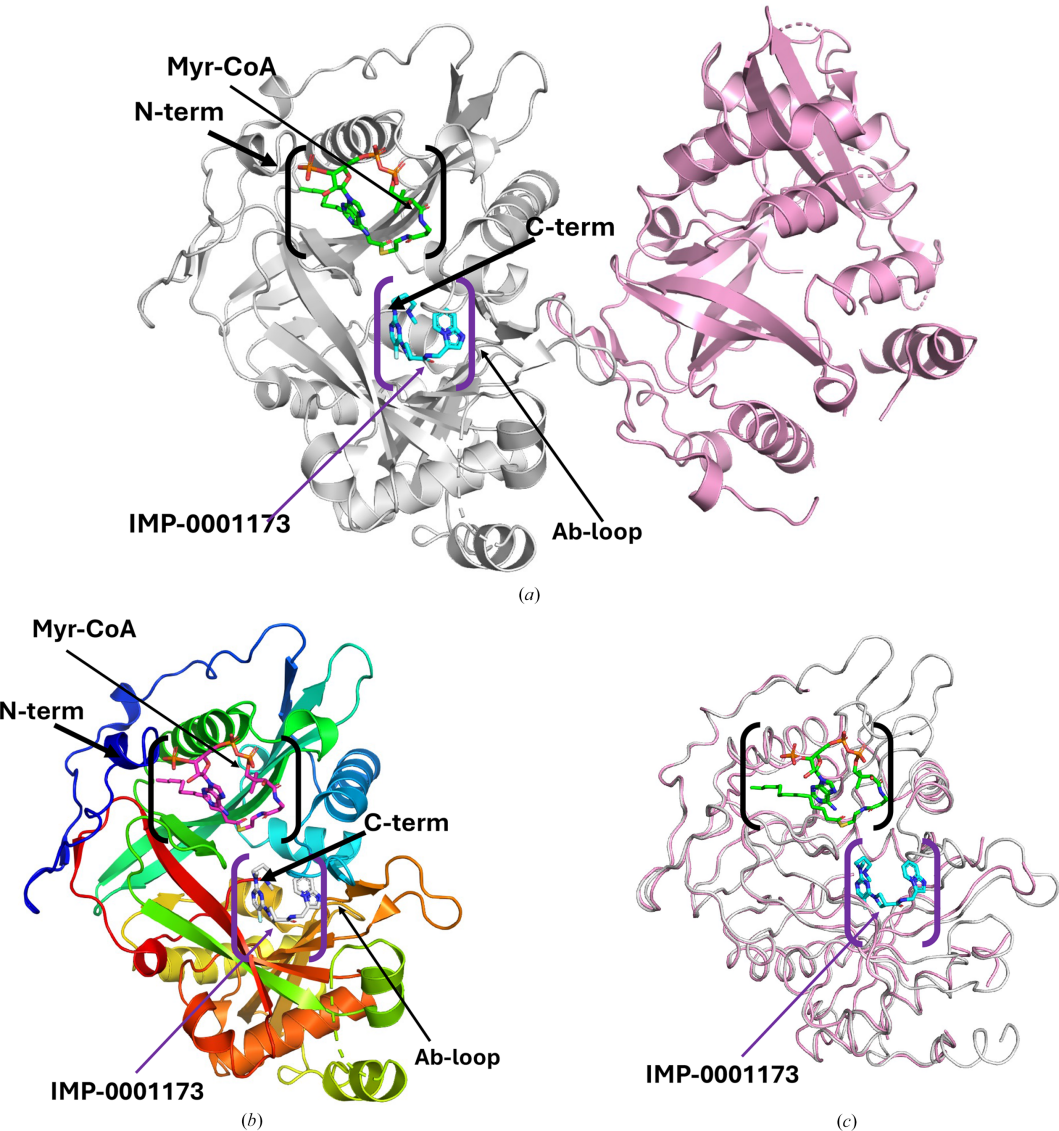
*Pv*NMT structure. (*a*) There are two *Pv*NMT monomers in the asymmetric unit. Chain *A* (gray) has a bound Myr-CoA (green sticks) and inhibitor IMP-0001173 (blue sticks). Chain *B* (pink) shows a *Pv*NMT monomer in the apo state. (*b*) Cartoon of *Pv*NMT monomer *A* colored in a rainbow from blue at the N-terminus to red at the C-terminus. Myr-CoA (magenta sticks) and the inhibitor IMP-0001173 (white sticks) are shown. (*c*) Superposition of the monomers chain *A* (with ligands, gray) and chain *B* (apo *Pv*NMT, pink). The substrate-binding cavity is indicated in purple parentheses, while the Myr-CoA-binding cavity is shown in black parentheses.

The co-crystal structure of *Pv*NMT with a cofactor (Myr-CoA) and a peptide-binding-domain inhibitor (IMP-0001173) was determined at 2.3 Å resolution. Interestingly, the asymmetric unit contains two copies of *Pv*NMT: an apo *Pv*NMT and a ternary complex with IMP-0001173 and Myr-CoA (Fig. 1 ▸*a*). Analysis with the *Protein Interfaces, Surfaces and Assemblies* service *PISA* at the European Bioinformatics Institute (https://www.ebi.ac.uk/pdbe/prot_int/pistart.html) agrees with the SEC information that *Pv*NMT is a biological monomer. *Pv*NMT has a prototypical NMT topology and adopts a compact, spherical configuration consisting of 15 α-helices and 19 β-sheets (Supplementary Fig. S2). The N-terminal catalytic center has two distinct binding pockets that are responsible for substrate-binding and catalytic activities (Fig. 1 ▸*b*). The substrate-binding pocket specifically binds the N-terminal sequence of myristoylated proteins, while a second proximal pocket acts as the cofactor-binding site (Fig. 1 ▸*b*). In our ternary structure, the substrate-binding pocket contains the inhibitor IMP-0001173 molecule, while the Myr-CoA molecule sits in the cofactor-binding site (Figs. 1 ▸*a* and 1 ▸*b*). Also evident from our structure is the location of the Ab-loop (Fig. 1 ▸*b*). This binding arrangement facilitates the transfer of myristic acid from Myr-CoA to the N-terminus of the substrate protein, resulting in the release of CoA as a byproduct of the reaction (Rudnick *et al.*, 1993 ▸; Spassov *et al.*, 2023 ▸).

The carboxyl-terminus of *Pv*NMT is inaccessible and positioned deep within the protein core (Figs. 1 ▸*a* and 1 ▸*b*), which ensures its resistance to cleavage by carboxypeptidases (Rudnick *et al.*, 1993 ▸). *Pv*NMT has a central core with an internal pseudo-twofold symmetry axis formed by the N-terminal and C-terminal halves, shaping the site for binding peptide substrates. All of the loops near the binding cavity are ordered in the monomer with bound Myr-CoA and IMP-0001173, whereas the loops nearest the binding cavities are disordered in the apo *Pv*NMT monomer (Fig. 1 ▸*c*). The partly ordered apo *Pv*NMT is the only reported apo *Pv*NMT structure in the PDB.

**Figure 2:**
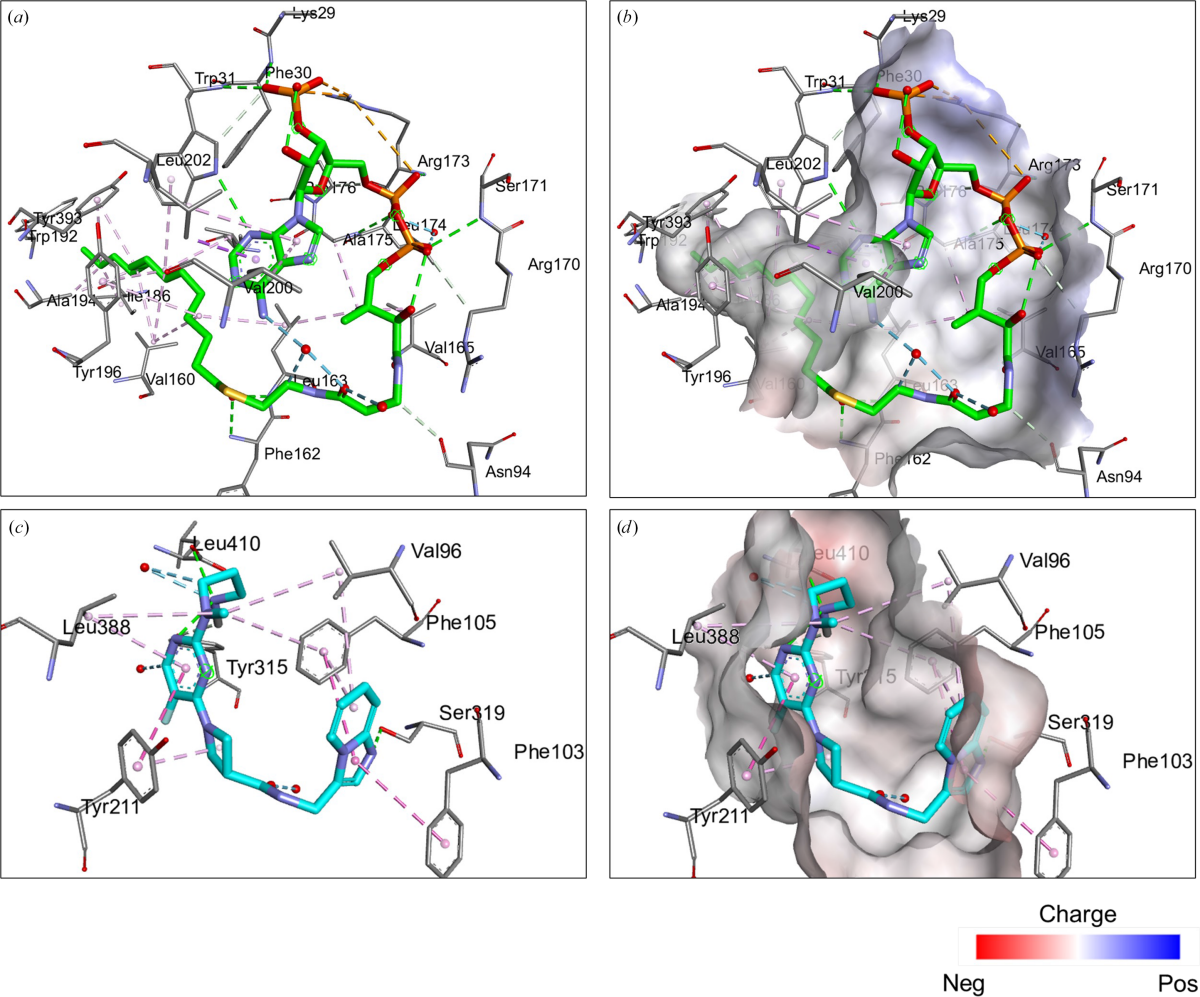
Myr-CoA and inhibitor (IMP-0001173) binding by *Pv*NMT. The interactions were analyzed with *Discovery Studio Visualizer* (https://discover.3ds.com/discovery-studio-visualizer). (*a*) Stick representation of amino-acid residues (gray) interacting with Myr-CoA (green). (*b*) The Myr-CoA-binding pocket is slightly positively charged [shown in the same view as in (*a*)]. (*c*) The inhibitor IMP-0001173 (shown in cyan) interacts with amino acids (gray). (*d*) IMP-0001173 has primarily electrostatic interactions in the peptide-binding pocket.

**Figure 3:**
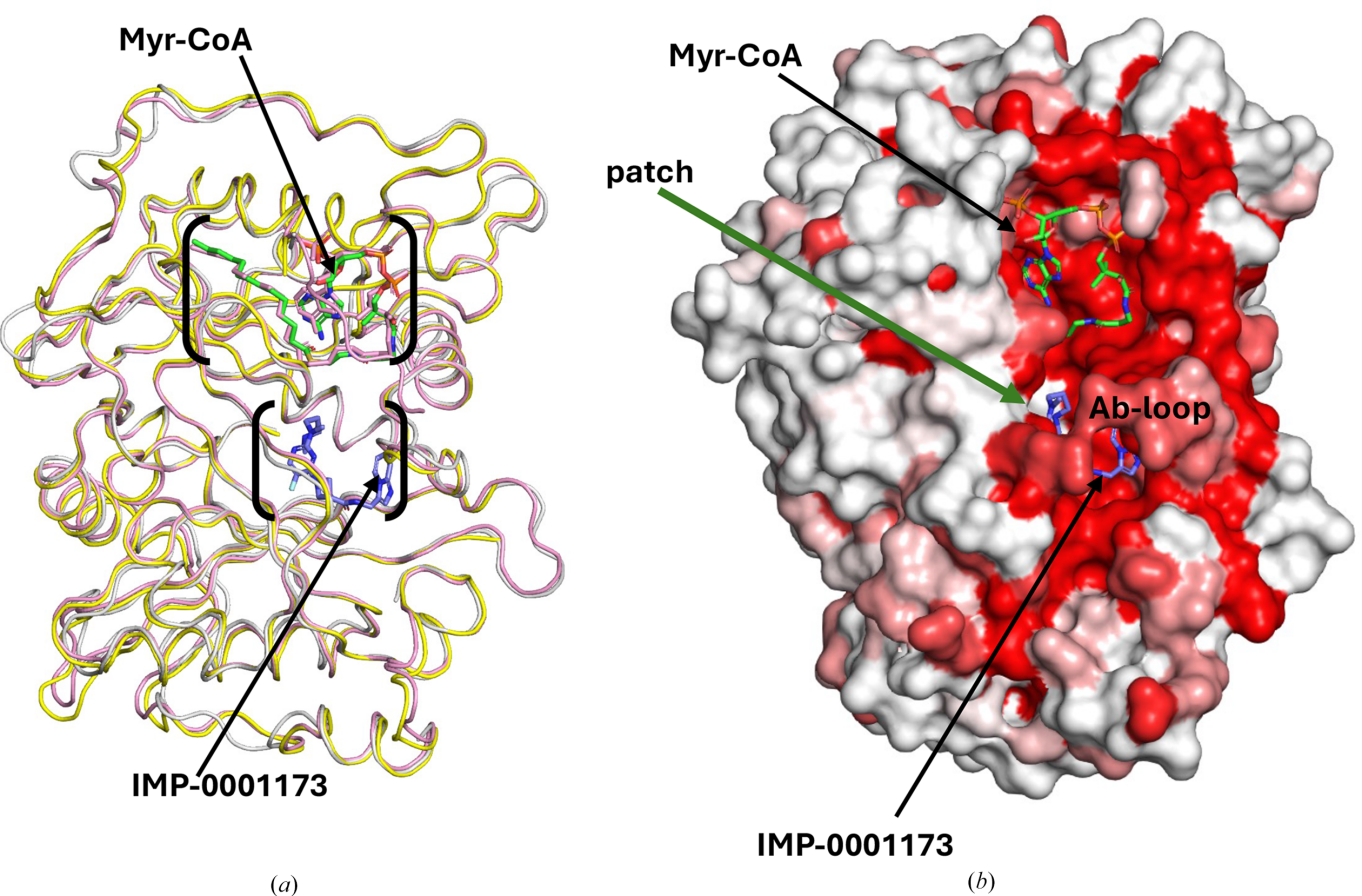
Comparison of human NMTs with *Pv*NMT. (*a*) *Pv*NMT (gray) shares structural topology with the two human NMTs *Hs*NMT1 (yellow) and *Hs*NMT2 (pink). Myr-CoA is shown in green sticks, while IMP-0001173 is shown in blue sticks. (*b*) An *ENDScript* surface plot of *Pv*NMT, in the same orientation as in (*a*), shows that the residues closest to the Myr-CoA- and peptide-binding sites are highly conserved and form an interconnected cavity. Red regions represent higher conserved regions, while white represents regions with low conservation. Myr-CoA is shown in green sticks while IMP-0001173 is shown in blue sticks.

**Figure 4:**
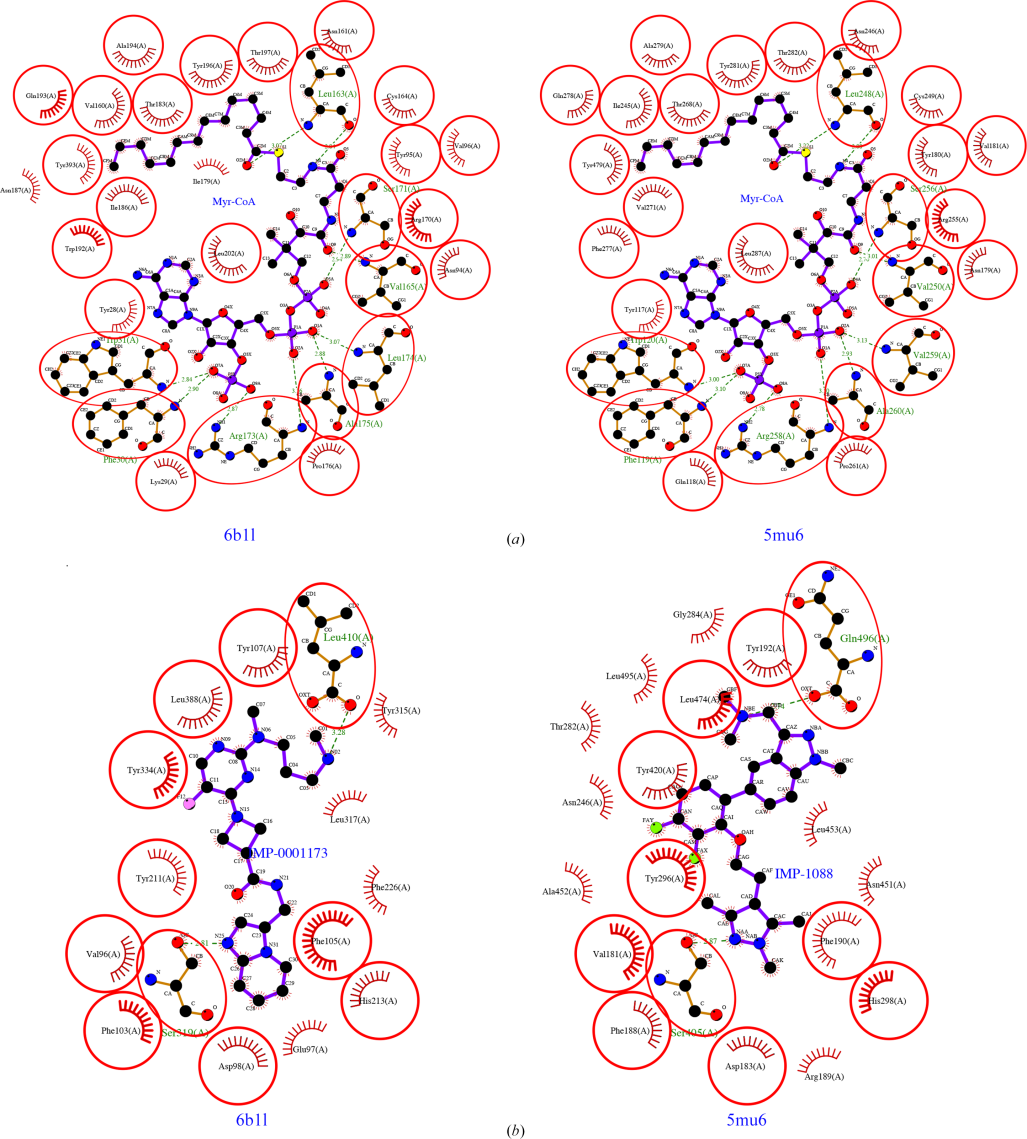
*LIGPLOT*-generated interaction plots for *Pv*NMT (PDB entry 6b1l) and *Hs*NMT1 (PDB entry 5mu6) reveal (*a*) conserved Myr-CoA cavities and (*b*) differences in the peptide-binding cavity. The *Hs*NMT1 structure (PDB entry 5mu6) has IMP-1088 within the peptide-binding pocket compared with IMP-0001173 in the *Pv*NMT structure (PDB entry 6b1l).

The myristoyl-binding pocket of *Pv*NMT preferentially binds glycine residues, ensuring its substrate specificity (Harupa *et al.*, 2020 ▸). In our structure, Myr-CoA occupies the extended groove that runs across one face of the enzyme (Fig. 2 ▸). Several leucine residues are involved in Myr-CoA binding (Rodríguez-Hernández *et al.*, 2023 ▸). The predominantly hydrophobic Myr-CoA binding site has a few positive charges that stabilize Myr-CoA binding (Harupa *et al.*, 2020 ▸; Rodríguez-Hernández *et al.*, 2023 ▸). The structures of complexes of NMTs from different organisms with substrates, intermediate stages, inhibitors and products have helped to clarify the catalytic mechanisms of NMT (Rodríguez-Hernández *et al.*, 2023 ▸; Wu *et al.*, 2007 ▸). The postulated catalytic mechanism starts with the formation of a stable complex with Myr-CoA, while the peptide-binding domain of NMT accepts the N-terminus of the substrate protein (Dian *et al.*, 2020 ▸; Rodríguez-Hernández *et al.*, 2023 ▸; Wu *et al.*, 2007 ▸). The Ab-loop above the peptide-binding pocket (Figs. 1 ▸*a*, 1 ▸*b* and 3 ▸*a*) adopts an closed or open conformation to control access to the active site by forming a ceiling or lid (Wu *et al.*, 2007 ▸). In our structure, the Ab-loop loop closes around the bound inhibitor (Fig. 3 ▸*b*). Opening of the Ab-loop allows initial peptide binding and subsequent release of the myristoylated peptide (Wu *et al.*, 2007 ▸). The surface plot shows the interconnectedness of the binding pockets of *Pv*NMT (Fig. 3 ▸*b*). In our *Pv*NMT ternary structure, the inhibitor IMP-0001173 is in the peptide-binding pocket, with a similar conformation as observed in other plasmodial NMT structures (Figs. 2 ▸ and 3 ▸). The peptide-binding pocket is predominantly hydrophobic but has some hydrogen bonds and salt bridges (Fig. 4 ▸*b*). An N atom (N02) of IMP-0001173 forms a salt bridge with the C-terminal carboxylate group (Leu410) of *Pv*NMT that effectively abrogates myristate transfer (Figs. 2 ▸ and 4 ▸*b*), making IMP-0001173 an effective inhibitor.

Structure-based primary-sequence alignment of *Pv*NMT with human NMTs (*Hs*NMT1 and *Hs*NMT2) and other plasmodial NMTs reveals a shared similarity of plasmodial NMTs and greater divergence from human NMTs (Supplementary Fig. S3). Nonetheless, the overall structural similarity between human NMTs and *Pv*NMT is evident from superposed representative structures (Fig. 3 ▸*a*). The representative human NMT structures are PDB entries 5mu6 for *Hs*NMT1 (Mousnier*et al.*, 2018 ▸) and 4c2x for *Hs*NMT2 (Thinon *et al.*, 2014 ▸). *ENDScript* (Gouet *et al.*, 2003 ▸; Robert & Gouet, 2014 ▸) analysis was used to identify the closest structural neighbors of *Pv*NMT (Supplementary Fig. S4). These analyses reveal that *Pv*NMT shares significant secondary-structural similarity with several NMTs, with identical residues observed across both the Myr-CoA- and peptide-binding domains (Supplementary Fig. S4). The regions of highest similarity are in the interconnected Myr-CoA- and peptide-binding cavities and are shown in red on the surface diagram (Fig. 3 ▸*b*). Interestingly, there is a patch of white in the peptide-binding cavity (Fig. 3 ▸*b*). Further details of structural differences and similarities are indicated in the sausage plot which, like the surface plot, was generated with *ENDScript* (Supplementary Fig. S5). The sausage plot shows the well conserved tertiary-structure topology in the protein core (thin sausages).

The differences between *Pv*NMT and human NMTs are being explored for drug discovery (Rodríguez-Hernández *et al.*, 2023 ▸; Harupa *et al.*, 2020 ▸). These differences are evident in *LIGPLOT*-generated (Laskowski & Swindells, 2011 ▸; Wallace *et al.*, 1995 ▸) interaction plots. Comparing our ternary structure of *Pv*NMT with that of human NMT (*Hs*NMT1) with an inhibitor of similar family as IMP-0001173, IMP-1088 (Bell *et al*., 2012 ▸; Mousnier *et al.*, 2018 ▸), reveals a well conserved Myr-CoA and differences in the peptide-binding domain (Fig. 4 ▸, Supplementary Table S1). Thus, *Pv*NMT is attractive for the rational development of small-molecule inhibitors due to differences in its peptide-binding domain from those of human NMTs (Rodríguez-Hernández *et al.*, 2023 ▸; Harupa *et al.*, 2020 ▸).

## 4. Conclusion

The presented *Pv*NMT ternary structure offers additional insights for the rational design and optimization of NMT inhibitors for the treatment of *P. vivax* malaria. Efforts are ongoing to translate these insights into future therapeutic interventions.

## Supplementary Material

PDB reference: *P. vivaxN*-myristoyltransferase, complex with myristoyl-CoA and IMP-0001173, 6b1l

Supplementary Figures and Table. DOI: 10.1107/S2053230X24008604/ir5034sup1.pdf

## Figures and Tables

**Table 1:** Macromolecule-production information

Source organism	*Plasmodium vivax* Sal-1 (strain Salvador I)
DNA source	GenScript gene synthesis
Expression vector	pET-11a (GenScript)
Expression host	*Escherichia coli* BL21(DE3) Rosetta
Complete amino-acid sequence of the construct produced	MGSSHHHHHHSAALEVLFQGPDYKFWYTQPVPKINDEFNESVNEPFISDNKVEDVRKDEYKLPPGYSWYVCDVKDEKDRSEIYTLLTDNYVEDDDNIFRFNYSAEFLLWALTSPNYLKTWHIGVKYDASNKLIGFISAIPTDICIHKRTIKMAEVNFLCVHKTLRSKRLAPVLIKEITRRINLENIWQAIYTAGVYLPKPVSDARYYHRSINVKKLIEIGFSSLNSRLTMSRAIKLYRVEDTLNIKNMRLMKKKDVEGVHKLLGSYLEQFNLYAVFTKEEIAHWFLPIENVIYTYVNEENGKIKDMISFYSLPSQILGNDKYSTLNAAYSFYNVTTTATFKQLMQDAILLAKRNNFDVFNALEVMQNKSVFEDLKFGEGDGSLKYYLYNWKCASFAPAHVGIVLL

**Table 2:** Crystallization

Method	Vapor diffusion, sitting drop
Plate type	96-well plates
Temperature (K)	290
Protein concentration (mg ml^−1^)	13.5
Buffer composition of protein solution	20 m*M* HEPES pH 7.0, 300 m*M* NaCl, 5%(*v*/*v*) glycerol, 1 m*M* TCEP, 0.4 m*M* Myr-CoA, 0.4 m*M* IMP-0001173
Composition of reservoir solution	0.06 *M* magnesium chloride hexahydrate, 0.06 *M* calcium chloride dihydrate, 0.1 *M* Tris–Bicine pH 8.5, 19.6%(*v*/*v*) PEG 500 MME, 9.8%(*w*/*v*) PEG 20 000
Volume and ratio of drop	0.4 µl, 1:1
Volume of reservoir (µl)	80
Composition of cryoprotectant solution	20%(*v*/*v*) ethylene glycol, 0.06 *M* magnesium chloride hexahydrate, 0.06 *M* calcium chloride dihydrate, 0.1 *M* Tris–Bicine pH 8.5, 19.6%(*v*/*v*) PEG 500 MME, 9.8%(*w*/*v*) PEG 20 000

**Table 3:** Data collection and processing Values in parentheses are for the outer shell.

Diffraction source	Beamline 5.0.2, ALS
Wavelength (Å)	1.00
Temperature (K)	100
Detector	Dectris PILATUS3 6M
Crystal-to-detector distance (mm)	475
Rotation range per image (°)	0.25
Total rotation range (°)	150
Space group	*P*2_1_2_1_2
*a*, *b*, *c* (Å)	80.03, 81.49, 119.44
α, β, γ (°)	90, 90, 90
Resolution range (Å)	48.17–2.30 (2.36–2.30)
Total No. of reflections	189923
No. of unique reflections	35084
Completeness (%)	99.10 (92.30)
Multiplicity	5.41 (4.79)
〈*I*/σ(*I*)〉	19.19 (3.03)
*R*_r.i.m._	0.065 (0.58)
Overall *B* factor from Wilson plot (Å^2^)	40.53

**Table 4:** Structure solution and refinement Values in parentheses are for the outer shell.

Resolution range (Å)	48.17–2.30 (2.36–2.30)
Completeness (%)	99.0 (92.3)
σ Cutoff	*F* > 1.34σ(*F*)
No. of reflections, working set	35070 (2327)
No. of reflections, test set	1884 (144)
Final *R*_cryst_	0.232 (0.272)
Final *R*_free_	0.271 (0.315)
No. of non-H atoms	
Protein	5264
Ligand	94
Solvent	188
Total	5546
R.m.s. deviations	
Bond lengths (Å)	0.002
Angles (°)	0.543
Average *B* factors (Å^2^)	
Protein	58.0
Ligand	42.9
Water	46.0
Ramachandran plot	
Most favored (%)	96
Allowed (%)	4
