# Supplementary material for: Ternary structure of Plasmodium vivaxN-myristoyltransferase with myristoyl-CoA and inhibitor IMP-0001173
Source: Acta Crystallogr F Struct Biol Commun. 2024 Sep 18;80(Pt 10):269–77. doi: 10.1107/S2053230X24008604 (PMC11448930; doi:10.1107/S2053230X24008604)
Supplement: Supplementary file 1 [file f-80-00269-sup1.pdf]

STRUCTURAL BIOLOGY COMMUNICATIONS

Volume 80 (2024)

Supporting information for article:

Ternary structure of *Plasmodium vivax N*-myristoyltransferase with myristoyl-CoA and inhibitor IMP-0001173

Cydni Bolling, Alex Mendez, Shane Taylor, Stanley Makumire, Alexandra Reers, Rachael Zigweid, Sandhya Subramanian, David M. Dranow, Bart Staker, Thomas E. Edwards, Edward W. Tate, Andrew S. Bell, Peter J. Myler, Oluwatoyin A. Asojo and Graham Chakafana

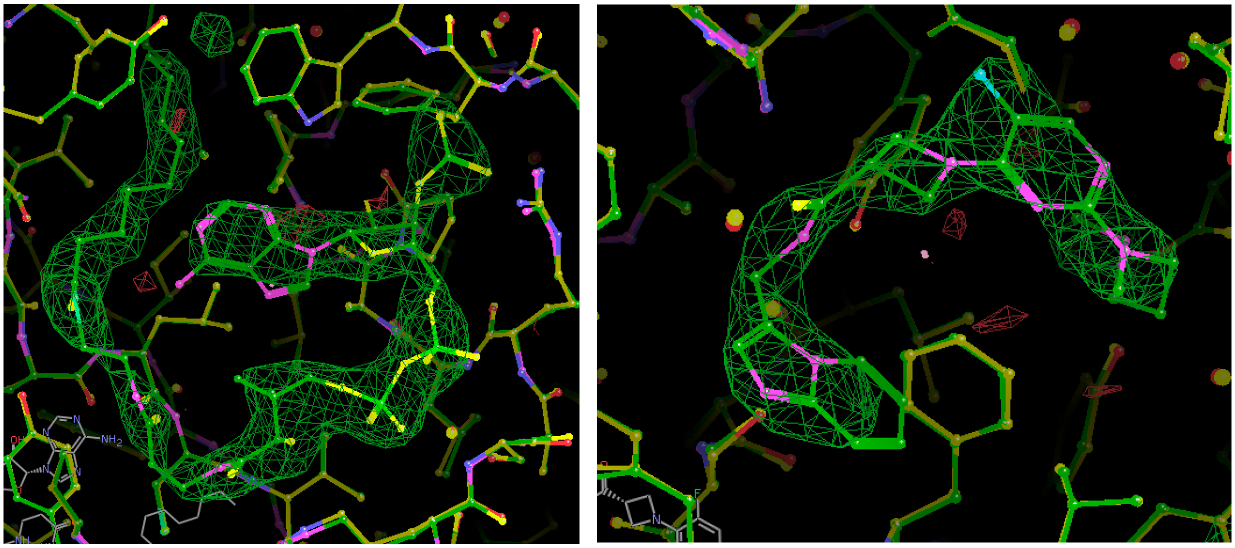

(a) (b)

Figure S1 Difference (Fo-Fc) omit electron density maps (green mesh) contoured at 3.5 sigma revealed 2 large blobs that matched the superposed ligands (shown in stick). (a) The Myr-CoA molecule fits into the electron density, as does (b) the inhibitor IMP-0001073 molecule.

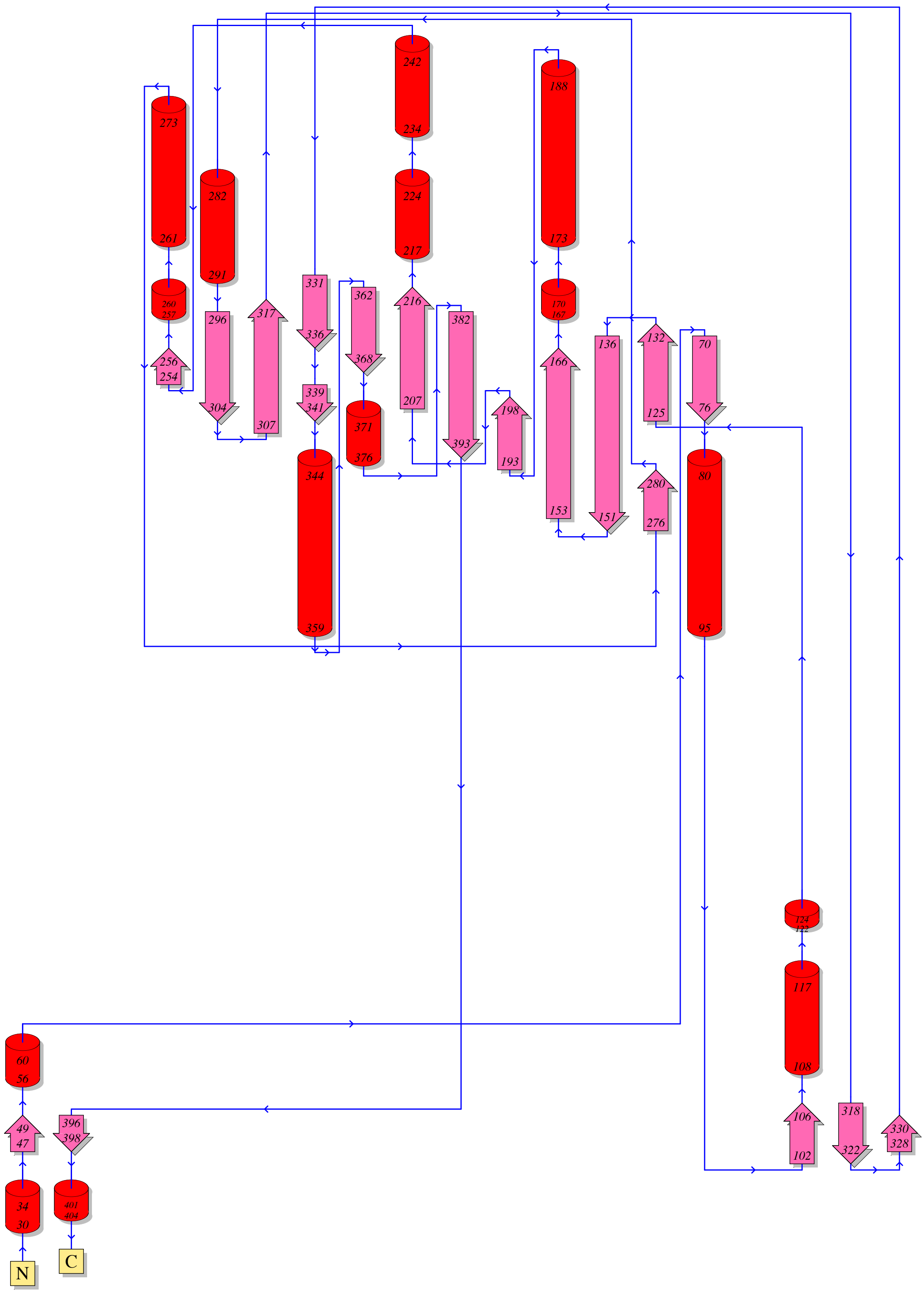

Figure S2. *Pv*NMT overall topology

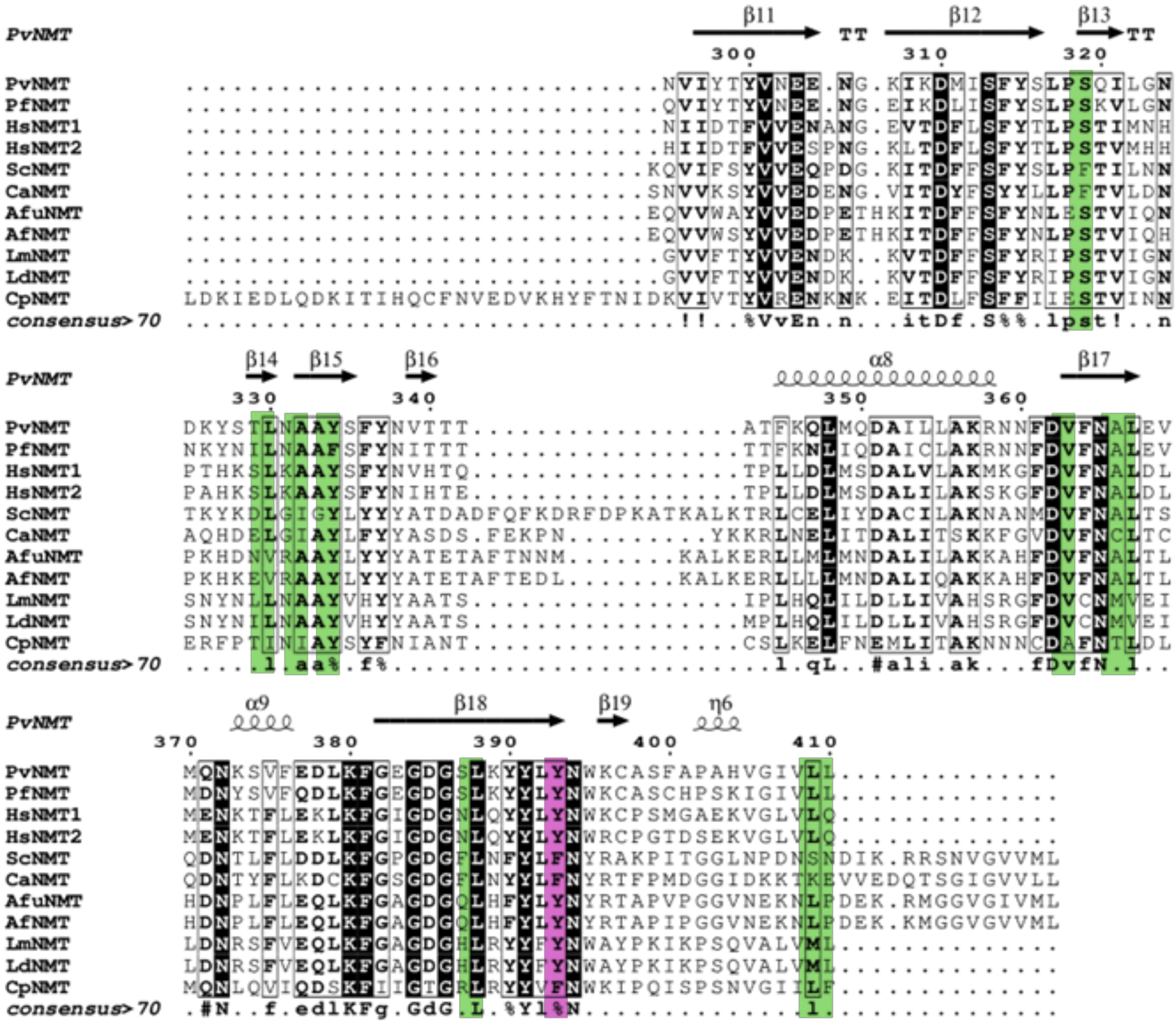

Figure S3. Comparison of plasmodial NMTs with human orthologues. NMT sequences used are from *P. vivax* (A0A1G4H3M1), *P. falciparum* (Q8ILW6), *P. yoelii* (Q7RPB1), *P. knowlesi* (A0A384LCS5) and the human NMT1 (P30419) and NMT2 (O60551). *The specific amino acids involved in inhibitor binding are indicated in Table S1. Identical residues are shaded black, while conserved ones are in gray.

Figure S4. ENDSCRIPT analysis reveals the nearest structural neighbors of *Pv*NMT and shows extensive sequence conservation across multiple organisms. Identical and conserved residues are highlighted in red and yellow, respectively. The different secondary structure elements shown are alpha helices (α), 3_{10}-helices (η), beta strands (β), and beta turns (TT).

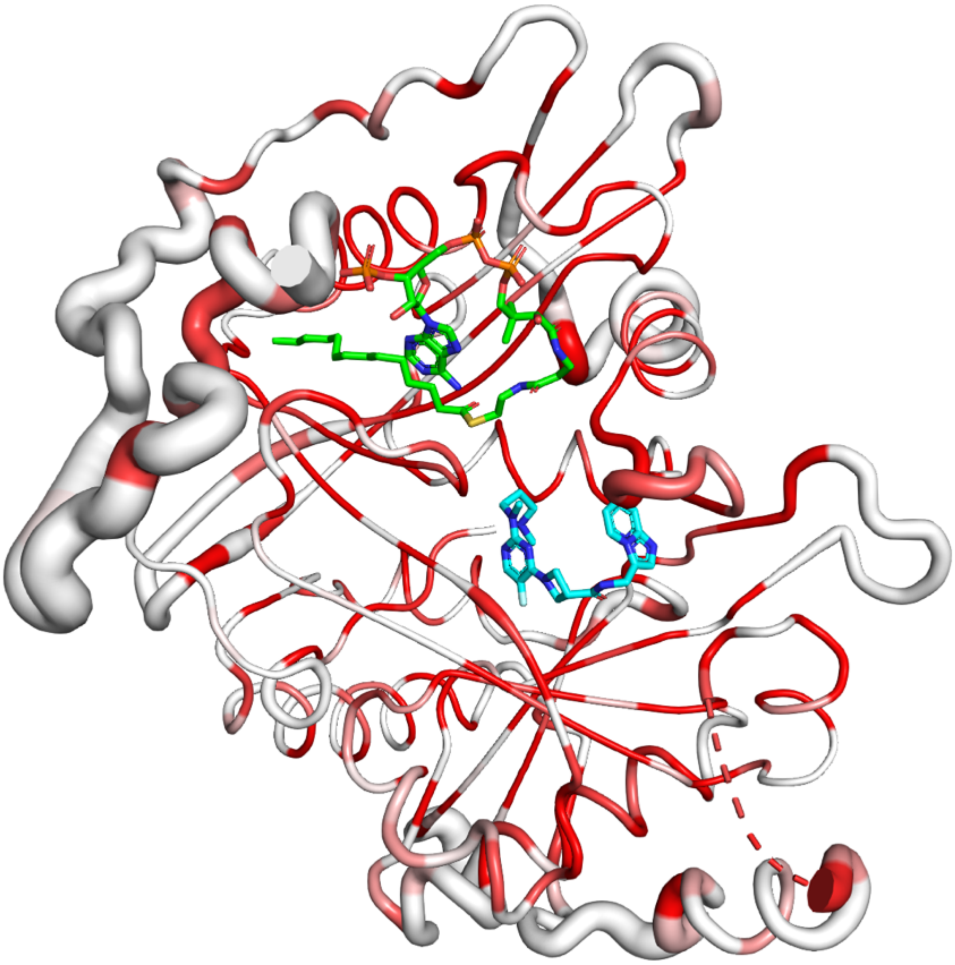

Figure S5. ENDSCRIPT analysis reveals structural variation in 3-D as sausage plots. Identical residues are indicated in red. The thicker sausage indicates higher tertiary structure differences. This figure allows us to visualize sequence identity and tertiary structure similarity simultaneously. In some regions, sequence identity is low (white), and tertiary structure similarity is high (thin sausages). In others, amino acids are identical (red), and tertiary structure similarity is low (fat sausages). Other combinations are also possible with sequence identity ranging from identical (red) to conserved (pink) to completely different (white). IMP-0001173 is indicated in blue, while Myr-CoA is in green sticks.

Table S1. Comparative analysis of residues involved in inhibitor and Myr-CoA binding.

Protein	PDB ID	Cofactor binding pocket residues	Substrate binding pocket residues
*Pv*NMT	6B1L	Tyr28, Lys29, Asn94, Tyr95, Val96, Val160, Asn161, Cys164, Arg170, Pro176, Ile179, Thr183, Ile186, Asn187, Trp192, Gln193, Ala194, Tyr196, Thr197, Leu202, Tyr393	Val96, Glu97, Asp98, Phe103, Phe105, Tyr107, Tyr211, His213, Phe226, Tyr315, Leu317, Leu388
HNMT1	5MU6	Tyr117, Gln118, Phe119, Trp120, Asn179, Tyr180, Val181, Ile245, Asn246, Leu248, Cys249, Val250, Leu254, Arg255, Ser256, Lys257, Arg258, Val259, Ala260, Pro261, Thr268, Val271, Phe277, Gln278, Ala279, Tyr281, Thr282, leu287, Tyr479	Val181, Asp183, Phe188, Arg189, Phe190, Tyr192, Asn246, Thr282, Gly284, His298, Tyr296, Ser405, Tyr420, Asn451, Ala452, Leu453, Leu474, Leu495, Gln496
